# Empirical analysis of adversarial robustness in 3D Gaussian Splatting under multi-view inconsistency attacks

**DOI:** 10.1038/s41598-026-53843-3

**Published:** 2026-06-01

**Authors:** Hyun Kwon, Jang-Woon Baek

**Affiliations:** 1 Department of Artificial Intelligence and Data Science, Korea Military Academy, Seoul, 01805 South Korea; 2 Department of Architectural Engineering, Kyung Hee University, Yongin Gyeonggi, 17104 South Korea

**Keywords:** 3D Gaussian Splatting, Adversarial robustness, Novel view synthesis, Multi-view reconstruction, Deep learning security, Engineering, Mathematics and computing

## Abstract

3D Gaussian Splatting has emerged as a promising technique for real-time novel view synthesis, achieving rendering quality comparable to neural radiance fields while enabling significantly faster inference. Despite its growing adoption in various applications including autonomous driving, robotics, and augmented reality, the adversarial robustness of 3D Gaussian Splatting remains largely unexplored. This paper presents a comprehensive empirical analysis of 3D Gaussian Splatting robustness against multi-view inconsistency attacks, which inject imperceptible perturbations into training images to disrupt the reconstruction process. We propose an adversarial attack framework that maximizes view-dependent inconsistencies while preserving visual imperceptibility through semantic-aware perturbation constraints. Our extensive experiments on the Mip-NeRF 360 dataset reveal that 3D Gaussian Splatting exhibits strong robustness to multi-view photometric inconsistency attacks, with quality degradation limited to less than one percent across standard metrics including Peak Signal-to-Noise Ratio, Structural Similarity Index, and Learned Perceptual Image Patch Similarity. We identify three key factors contributing to this robustness: multi-view averaging during optimization, the explicit Gaussian primitive representation, and the gradient-based optimization dynamics that naturally suppress view-dependent artifacts. These findings provide important insights for deploying 3D Gaussian Splatting in security-sensitive applications and suggest directions for developing more effective adversarial attack strategies.

## Introduction

The synthesis of novel viewpoints from a sparse set of input images has been a fundamental challenge in computer vision and graphics for decades. Recent advances in neural rendering have revolutionized this field, with Neural Radiance Fields demonstrating unprecedented quality in representing complex 3D scenes through implicit neural representations^1^. However, the computational demands of volumetric ray marching in neural radiance fields have limited their practical deployment in real-time applications. 3D Gaussian Splatting has emerged as a compelling alternative that achieves comparable rendering quality while enabling real-time performance through explicit 3D Gaussian primitives and efficient rasterization^2^.

The rapid adoption of 3D Gaussian Splatting across diverse application domains raises important questions about its reliability and security. In autonomous driving systems, 3D scene representations are used for simulation and sensor data augmentation^3,4^. Robotic manipulation systems rely on accurate 3D reconstructions for planning and control^5^. Augmented reality applications require robust scene understanding for seamless integration of virtual content^6^. In these safety-critical contexts, understanding the vulnerability of reconstruction algorithms to adversarial manipulation becomes essential.

Adversarial attacks on deep learning systems have been extensively studied in the context of image classification^7,8^, object detection^9^, and semantic segmentation^10^. These attacks demonstrate that imperceptible perturbations to input data can cause catastrophic failures in neural network predictions. More recently, researchers have begun investigating adversarial vulnerabilities in 3D vision systems, including point cloud classifiers^11^, depth estimation networks^12^, and neural radiance fields^13,14^.

Despite the growing body of work on adversarial attacks in 3D vision, the robustness of 3D Gaussian Splatting to such attacks remains unexplored. This gap in understanding is particularly significant given several unique characteristics of the 3D Gaussian Splatting framework. Unlike neural radiance fields that encode scene information in neural network weights, 3D Gaussian Splatting represents scenes through explicit 3D Gaussian primitives with learnable positions, covariances, colors, and opacities. This explicit representation may exhibit different vulnerability patterns compared to implicit neural representations. Furthermore, the optimization process in 3D Gaussian Splatting involves adaptive density control through splitting and pruning of Gaussians, which could potentially filter out inconsistencies introduced by adversarial perturbations.

This paper presents the first comprehensive study of adversarial robustness in 3D Gaussian Splatting. We focus on multi-view inconsistency attacks, which represent a natural threat model for multi-view reconstruction systems. In this attack paradigm, an adversary introduces view-dependent perturbations to training images that violate the multi-view consistency assumption underlying 3D reconstruction. Such attacks could be realized in practice through compromised camera sensors, manipulated training data, or adversarial patches in the physical scene.

The contributions of this paper are threefold. First, we propose an adversarial attack framework specifically designed for 3D Gaussian Splatting that maximizes multi-view inconsistency while maintaining perturbation imperceptibility through semantic-aware constraints. Second, we conduct extensive empirical evaluation on challenging real-world scenes from the Mip-NeRF 360 dataset, providing quantitative analysis of reconstruction quality degradation under attack. Third, we identify and analyze the factors contributing to the observed robustness of 3D Gaussian Splatting, offering insights that inform both the development of more effective attacks and the design of robust reconstruction systems.

Our experimental results reveal a surprising finding: 3D Gaussian Splatting demonstrates significant inherent robustness to multi-view inconsistency attacks. Despite successfully injecting view-dependent perturbations with high imperceptibility, the resulting degradation in reconstruction quality is minimal, with average decreases of 0.24 percent in Peak Signal-to-Noise Ratio, 0.68 percent in Structural Similarity Index, and 3.7 percent increase in Learned Perceptual Image Patch Similarity. These results suggest that the explicit Gaussian representation and the multi-view optimization process provide natural defenses against certain classes of adversarial attacks.

The remainder of this paper is organized as follows. Section “Related work” reviews related work on 3D Gaussian Splatting, adversarial attacks in computer vision, and security of 3D reconstruction systems. Section “Proposed method” presents our proposed multi-view inconsistency attack framework with detailed mathematical formulations. Section “Experiments” describes the experimental setup including datasets, implementation details, and evaluation metrics. Section “Experimental results” presents and analyzes our experimental results. Section “Discussion” discusses the implications of our findings and limitations of the current study. Section “Conclusion” concludes the paper with suggestions for future research directions.

## Related work

### Neural radiance fields and novel view synthesis

Neural Radiance Fields introduced a paradigm shift in novel view synthesis by representing scenes as continuous volumetric functions parameterized by neural networks^1^. Given a 5D input consisting of spatial location and viewing direction, the network outputs volume density and view-dependent radiance, which are integrated along camera rays to produce rendered images. This approach achieved unprecedented quality in novel view synthesis but required extensive computation for both training and rendering.

Subsequent research has focused on improving the efficiency and quality of neural radiance fields. Instant-NGP introduced multi-resolution hash encoding to dramatically accelerate training and rendering^15^. Mip-NeRF addressed aliasing artifacts through integrated positional encoding over conical frustums^16^, while Mip-NeRF 360 extended this approach to unbounded scenes with specialized parameterization and regularization^17^. Plenoxels demonstrated that explicit voxel-based representations could achieve competitive quality without neural networks^18^. TensoRF factorized the radiance field into compact tensor components for efficient representation^19^.

The development of 3D Gaussian Splatting represents a significant advancement in explicit scene representations^2^. By representing scenes as collections of 3D Gaussian primitives with learnable parameters, this approach achieves real-time rendering through efficient tile-based rasterization while maintaining quality comparable to state-of-the-art neural radiance field methods. The explicit nature of the representation enables direct manipulation and editing of scene content^20^, dynamic scene modeling^21,22^, and efficient compression^23^.

### Adversarial attacks in computer vision

Adversarial attacks on deep neural networks were first systematically studied in the context of image classification^7,24^. The Fast Gradient Sign Method provided an efficient approach for generating adversarial perturbations by following the sign of the loss gradient^7^. Projected Gradient Descent extended this approach through iterative optimization with perturbation constraints^8^. The Carlini and Wagner attack formulated adversarial example generation as an optimization problem with carefully designed objective functions^25^.

Adversarial attacks have been extended to various computer vision tasks beyond classification. In object detection, adversarial patches can cause detectors to miss objects or produce false positives^9,26^. Semantic segmentation networks are vulnerable to perturbations that cause systematic misclassification of scene regions^10,27^. Depth estimation networks can be attacked to produce incorrect depth predictions with implications for autonomous navigation^12,28^.

The study of adversarial robustness has also motivated the development of defense mechanisms. Adversarial training incorporates adversarial examples into the training process to improve model robustness^8,29^. Certified defenses provide provable guarantees against perturbations within specified bounds^30,31^. Input preprocessing techniques attempt to remove adversarial perturbations before classification^32,33^.

Physical-world adversarial attacks on 3D vision systems have received growing attention beyond classification and detection. Cheng et al.^34^ demonstrated that optimization-based adversarial patches can effectively attack monocular depth estimation in autonomous driving scenarios, achieving significant depth estimation errors while maintaining patch imperceptibility. Extending this line of work, Cheng et al.^35^ showed that camera-LiDAR fusion models, commonly regarded as inherently robust due to multi-modal redundancy, remain vulnerable to single-modal camera-only adversarial attacks, challenging the assumption that sensor fusion provides a general defense. On the defense side, Cheng et al.^36^ proposed a self-supervised adversarial training framework for monocular depth estimation that hardens models against physical-world attacks without requiring ground-truth depth labels, leveraging view synthesis consistency as a training signal. These works collectively highlight that adversarial vulnerabilities in 3D perception systems are not limited to implicit neural representations, and that targeted defense strategies must account for the specific properties of each representation type-a consideration directly relevant to our analysis of 3D Gaussian Splatting robustness.

### Security of 3D vision systems

The security of 3D vision systems has received increasing attention as these systems are deployed in safety-critical applications. Point cloud classifiers have been shown to be vulnerable to adversarial point perturbations, additions, and removals^11,37,38^. LiDAR-based perception systems in autonomous vehicles can be attacked through spoofing and adversarial object placement^39,40^.

Neural radiance fields present unique security considerations due to their data-driven training process. Adversarial attacks on neural radiance fields have been explored through perturbations to training images that degrade rendering quality^13^. NeRF-Attack demonstrated that targeted perturbations can cause neural radiance fields to render incorrect content at specific viewpoints^14^. Backdoor attacks on neural radiance fields have shown that hidden triggers can be embedded during training^41^.

The multi-view consistency assumption underlying 3D reconstruction provides a natural attack surface. Multi-view stereo systems rely on photometric consistency across views for depth estimation, which can be disrupted through view-dependent perturbations^42^. Structure from motion pipelines depend on feature matching across images, creating vulnerabilities to attacks that disrupt correspondence estimation^43^.

### Robustness analysis of reconstruction systems

Understanding the robustness of reconstruction systems extends beyond adversarial attacks to include natural corruptions and distribution shifts. Studies have examined the sensitivity of neural radiance fields to noisy camera poses^44^, varying image quality^6^, and sparse view inputs^45,46^. Gaussian splatting methods have been analyzed for their behavior under different initialization strategies and optimization hyperparameters^2^.

The relationship between representation choice and robustness has been explored in various contexts. Explicit representations such as voxel grids and point clouds may exhibit different failure modes compared to implicit neural representations^47^. Hybrid approaches that combine explicit and implicit components aim to leverage the advantages of both paradigms^48^.

Despite this growing body of work, a systematic analysis of 3D Gaussian Splatting robustness to adversarial attacks has not been conducted. This paper addresses this gap by providing the first comprehensive evaluation of 3D Gaussian Splatting under multi-view inconsistency attacks, contributing to our understanding of the security properties of this increasingly important representation.

## Proposed method

### Problem formulation

We consider the problem of attacking 3D Gaussian Splatting through adversarial perturbations to training images. Let ${\mathcal {I}} = \{I_1, I_2, \ldots , I_N\}$ denote the set of *N* training images capturing a scene from different viewpoints, with corresponding camera parameters ${\mathcal {C}} = \{C_1, C_2, \ldots , C_N\}$ obtained through structure from motion. The goal of 3D Gaussian Splatting is to optimize a set of 3D Gaussian primitives ${\mathcal {G}} = \{g_1, g_2, \ldots , g_M\}$ such that rendering from any training viewpoint closely matches the corresponding ground truth image.

Each Gaussian primitive $g_i$ is characterized by a mean position $\mu _i \in {\mathbb {R}}^3$, a covariance matrix $\Sigma _i \in {\mathbb {R}}^{3 \times 3}$ parameterized through scaling and rotation, spherical harmonic coefficients for view-dependent color, and an opacity value $\alpha _i \in [0, 1]$. The rendering process projects these 3D Gaussians onto the image plane and performs alpha blending to produce the final image.

Our attack objective is to generate adversarial images ${\mathcal {I}}^{adv} = \{I_1^{adv}, I_2^{adv}, \ldots , I_N^{adv}\}$ by adding perturbations $\delta _i$ to each training image such that the 3D Gaussian Splatting model trained on ${\mathcal {I}}^{adv}$ produces degraded rendering quality. We impose imperceptibility constraints on the perturbations to ensure that adversarial images remain visually indistinguishable from the originals.

### Multi-view inconsistency attack

The fundamental principle underlying our attack is the exploitation of multi-view consistency, which is the cornerstone of all multi-view 3D reconstruction methods. In a consistent multi-view setup, corresponding points across different views should exhibit similar appearance when accounting for viewpoint changes. By introducing view-dependent perturbations that violate this consistency, we aim to confuse the optimization process and degrade the quality of the reconstructed 3D Gaussians.

We formulate the multi-view inconsistency attack as an optimization problem. For a given set of training images and a pre-trained 3D Gaussian Splatting model, we seek perturbations that maximize the variance in rendered appearance across different viewpoints while maintaining perturbation imperceptibility. The overall objective function is defined as

1 $$\begin{aligned} {\mathcal {L}}_{total} = {\mathcal {L}}_{inc} + \lambda _{bg} {\mathcal {L}}_{bg} \end{aligned}$$

where ${\mathcal {L}}_{inc}$ is the inconsistency loss that encourages view-dependent variations, ${\mathcal {L}}_{bg}$ is the background preservation loss that focuses perturbations on foreground objects, and $\lambda _{bg}$ is a weighting coefficient.

### Inconsistency loss formulation

The inconsistency loss is designed to maximize the disagreement between rendered images from different viewpoints. Given a batch of *B* training viewpoints, we render the current 3D Gaussian model from each viewpoint and compute pairwise differences. The inconsistency loss is defined as

2 $$\begin{aligned} {\mathcal {L}}_{inc} = -\frac{1}{B(B-1)} \sum _{i=1}^{B} \sum _{j \ne i} \Vert R(I_i^{adv}) - R(I_j^{adv}) \Vert _2^2 \end{aligned}$$

where $R(I_i^{adv})$ denotes the rendered image from the *i*-th viewpoint after training on adversarial images. The negative sign indicates that we seek to maximize this disagreement through gradient ascent on the perturbations.

To make the optimization tractable, we approximate the effect of perturbations on the final rendering through a surrogate loss that directly operates on the perturbed training images. Specifically, we compute feature representations of the perturbed images using a pre-trained feature extractor and maximize the variance of these features across views. This surrogate formulation is given by

3 $$\begin{aligned} {\mathcal {L}}_{inc}^{surrogate} = -\text {Var}(\{F(I_1^{adv}), F(I_2^{adv}), \ldots , F(I_B^{adv})\}) \end{aligned}$$

where *F(· )* denotes feature extraction and $\text {Var}(\cdot )$ computes the variance across the batch.

### Background preservation

To create realistic attack scenarios and focus the attack on semantically meaningful regions, we incorporate background preservation into our framework. Real-world attacks are more likely to target specific objects of interest rather than entire scenes, and preserving background regions helps maintain the overall plausibility of the adversarial images.

We employ semantic segmentation to identify foreground objects and background regions. Let $M_i$ denote the binary mask for the *i*-th image, where $M_i(x, y) = 1$ indicates foreground pixels and $M_i(x, y) = 0$ indicates background pixels. The background preservation loss penalizes perturbations in background regions according to

4 $$\begin{aligned} {\mathcal {L}}_{bg} = \frac{1}{N} \sum _{i=1}^{N} \Vert (1 - M_i) \odot \delta _i \Vert _2^2 \end{aligned}$$

where *⊙* denotes element-wise multiplication. This loss encourages the optimization to concentrate perturbations on foreground objects while leaving background regions largely unchanged.

### Perturbation constraints

To ensure imperceptibility of the adversarial perturbations, we constrain the perturbation magnitude using both $L_\infty$ and $L_2$ bounds. The $L_\infty$ constraint limits the maximum change to any individual pixel value according to

5 $$\begin{aligned} \Vert \delta _i \Vert _\infty \le \epsilon \end{aligned}$$

where *ε* is the perturbation budget. We set $\epsilon = 16/255 \approx 0.0627$ following standard practices in adversarial attack literature.

The perturbation is applied to generate adversarial images through

6 $$\begin{aligned} I_i^{adv} = \text {clip}(I_i + \delta _i, 0, 1) \end{aligned}$$

where the clipping operation ensures that pixel values remain within the valid range.

### Optimization procedure

The adversarial perturbations are optimized using projected gradient descent. Starting from zero initialization, we iteratively update the perturbations by computing gradients of the total loss with respect to the perturbation variables and projecting the result onto the feasible set defined by the perturbation constraints.

At each iteration *t*, the update rule is given by

7 $$\begin{aligned} \delta _i^{(t+1)} = \Pi _\epsilon \left( \delta _i^{(t)} + \eta \cdot \text {sign}\left( \nabla _{\delta _i} {\mathcal {L}}_{total} \right) \right) \end{aligned}$$

where *η* is the step size, $\text {sign}(\cdot )$ extracts the sign of each gradient component, and $\Pi _\epsilon (\cdot )$ denotes projection onto the $L_\infty$ ball of radius *ε*.

The complete attack procedure processes training images in batches to enable computation of multi-view inconsistency. For each batch, we compute the inconsistency loss across views, the background preservation loss, and update the perturbations accordingly. The detailed procedure is presented in Algorithm 1.

**Algorithm 1:**
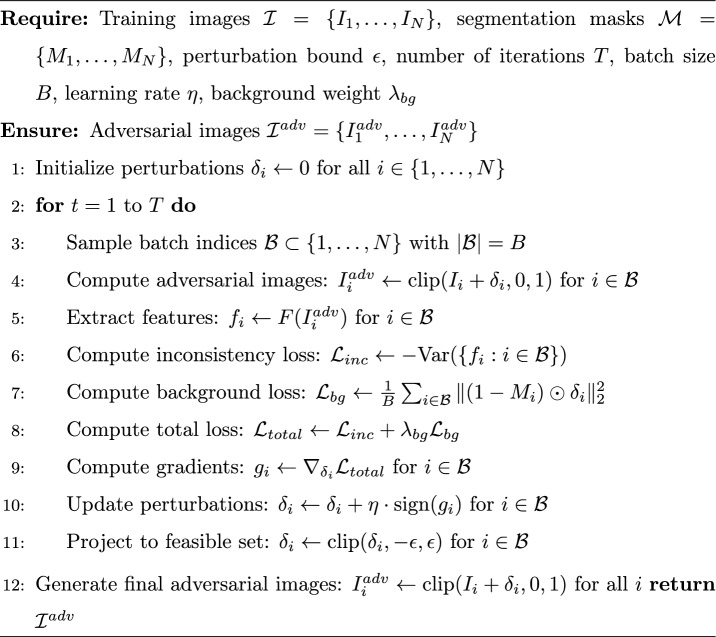
Multi-view inconsistency attack on 3D Gaussian Splatting

### Semantic segmentation for mask generation

The generation of foreground masks is accomplished using the Segment Anything Model, which provides class-agnostic segmentation of objects in images^49^. We employ Grounded-SAM, which combines the Segment Anything Model with text-grounded object detection to identify and segment specific object categories^50^.

For each training image, we provide text prompts describing the primary objects of interest in the scene and obtain corresponding segmentation masks. These masks are refined through morphological operations to remove small artifacts and ensure spatial coherence. The resulting masks guide the perturbation optimization to focus on semantically meaningful regions while preserving background content.

### Surrogate loss validation

To validate that the surrogate loss (Equation 3) is a reliable proxy for actual reconstruction degradation, we conducted a systematic experiment on the Garden scene by varying the perturbation budget $\epsilon \in \{4, 8, 16, 32, 64\}/255$ and measuring both the surrogate loss (feature variance) and the resulting PSNR, SSIM, and LPIPS degradation after full 3DGS training. Table 1 presents the results.

**Table 1:** Surrogate loss validation: correlation with actual degradation.

*ε*	Surrogate Loss	Absolute Values			Degradation (%)		
		PSNR	SSIM	LPIPS	*Δ*PSNR	*Δ*SSIM	*Δ*LPIPS
Baseline	-	27.41	0.858	0.122	-	-	-
4/255	2.00e-05	26.86	0.846	0.135	2.00	1.37	11.1
8/255	8.01e-05	26.90	0.846	0.136	1.87	1.35	11.6
16/255	3.20e-04	26.92	0.845	0.136	1.79	1.44	11.8
32/255	1.28e-03	26.87	0.844	0.137	1.99	1.58	12.6
64/255	5.13e-03	26.75	0.840	0.141	2.42	2.04	16.0
Pearson *r*		-			**0.934**	**0.995**	**0.996**
*p*-value		-			0.0201	0.0004	0.0003

**Fig. 1:**
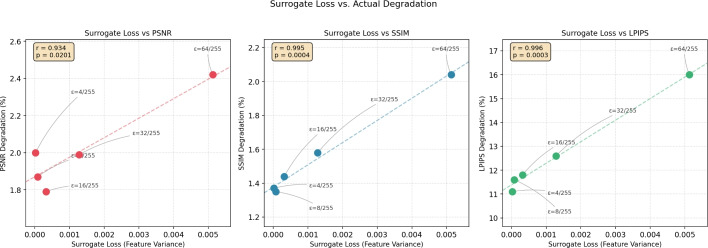
Scatter plots of surrogate loss (feature variance) versus actual degradation across five perturbation budgets ($\epsilon \in \{4,8,16,32,64\}/255$. Strong positive correlations (*r> 0.93*, *p < 0.05*) confirm that the surrogate effectively captures reconstruction inconsistency.

**Fig. 2:**
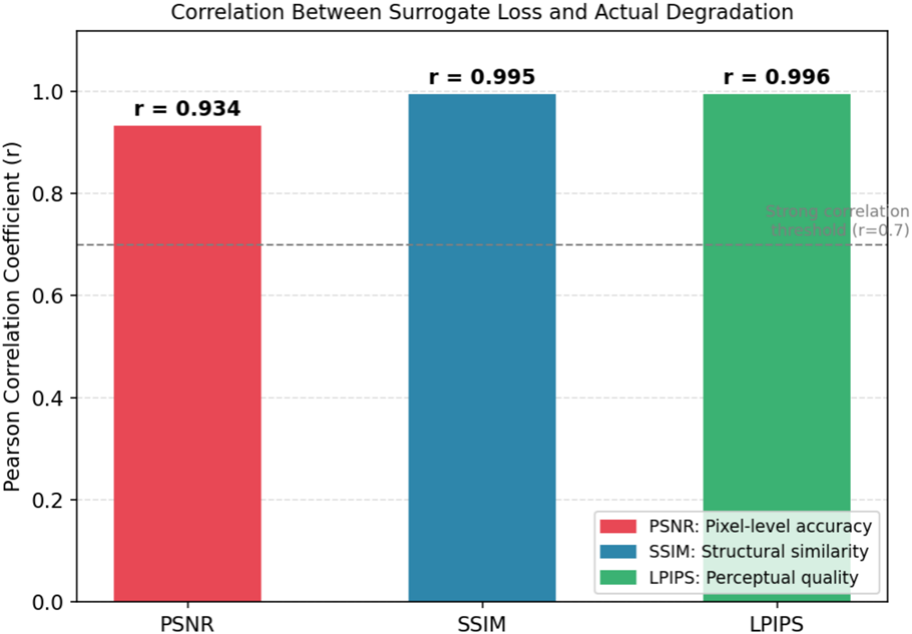
Pearson correlation coefficients between surrogate loss and actual degradation metrics. All values substantially exceed the strong-correlation threshold (*r = 0.7*), validating the surrogate approximation.

The surrogate loss exhibits strong positive correlation with all three degradation metrics: *r = 0.934* (*p = 0.020*) for PSNR, *r = 0.995* (*p < 0.001*) for SSIM, and *r = 0.996* (*p < 0.001*) for LPIPS, all substantially exceeding the conventional strong-correlation threshold of *r = 0.7*. As shown in Fig. 1, SSIM and LPIPS degradation increase monotonically with surrogate loss (*r> 0.99*), while PSNR shows a strong overall positive correlation (*r = 0.934*) with a non-monotonic variation in the intermediate *ε* range (*ε* = 4/255 to 16/255), which we attribute to stochastic noise inherent in 3DGS training optimization. Collectively, these results confirm that the surrogate loss is a valid proxy for actual reconstruction degradation. Figure 2 summarizes the correlation coefficients across metrics. These results empirically establish the validity of the surrogate approximation: the attack’s limited effectiveness reflects genuine 3DGS robustness rather than an approximation gap between the surrogate and the exact objective.

## Experiments

### Datasets

We evaluate our adversarial attack framework on scenes from the Mip-NeRF 360 dataset, which has become a standard benchmark for evaluating novel view synthesis methods on challenging real-world scenes^17^. This dataset contains unbounded outdoor and indoor scenes captured with high-resolution cameras, featuring complex geometry, diverse materials, and significant viewpoint variation.

We select five scenes spanning both outdoor and indoor environments for our experiments. For outdoor scenes, we use Garden and Bicycle from the original evaluation. For indoor scenes, we additionally include Room, Counter, and Kitchen, which feature distinct challenges including textureless surfaces, specular reflections, and limited viewpoint diversity compared to outdoor captures.

The Garden scene contains a table with various objects placed in a garden environment, captured from 185 training viewpoints at approximately 1600*×*1200 pixel resolution. The Bicycle scene depicts a bicycle in an outdoor setting, captured from 194 training viewpoints at similar resolution. The Room scene features indoor furniture with complex occlusion patterns. The Counter scene contains a kitchen counter with reflective surfaces and fine geometric detail. The Kitchen scene presents an indoor kitchen environment with high geometric complexity and varying lighting conditions. This diverse set of scenes enables analysis of robustness properties across different reconstruction challenges.

### Model configuration

We employ the official 3D Gaussian Splatting implementation as our target model^2^. The model is trained using the default hyperparameters specified in the original work, which have been shown to produce high-quality results across diverse scenes.

The initial point cloud for Gaussian initialization is obtained from structure from motion processing using COLMAP^51,52^. The optimization proceeds for 30,000 iterations, with the first 500 iterations dedicated to densification warm-up. Adaptive density control begins at iteration 500 and continues until iteration 15,000, with densification occurring every 100 iterations. The opacity reset is performed at iteration 3,000 to remove transparent Gaussians that contribute minimally to the rendering.

The learning rates follow an exponential decay schedule, with the position learning rate starting at 0.00016 and decaying to 0.0000016 by the end of training. The spherical harmonic coefficients are optimized with a learning rate of 0.0025, while opacity and scaling parameters use learning rates of 0.05 and 0.005 respectively.

For our attack, we configure the perturbation bound at 16/255, the number of attack iterations at 50, and the batch size at 8 views per iteration. The attack learning rate is set to 0.01, and the background preservation weight is set to 0.1. These parameters are selected to balance attack effectiveness with perturbation imperceptibility based on preliminary experiments.

### Experimental environment

All experiments are conducted on a workstation equipped with an NVIDIA RTX 4090 graphics processing unit with 24 gigabytes of video memory. The system contains an AMD EPYC 7313 processor and 512 gigabytes of DDR4 system memory. The software environment consists of Ubuntu 22.04 LTS operating system, PyTorch 2.0.1 deep learning framework, and CUDA 11.8 for GPU acceleration.

Semantic segmentation masks are generated using Grounded-SAM with the ViT-H backbone for the Segment Anything Model component. Text prompts are manually specified for each scene to identify the primary foreground objects. For the Garden scene, we use prompts targeting the table and objects placed on it. For the Bicycle scene, we target the bicycle as the primary foreground object.

We evaluate rendering quality using three standard metrics. Peak Signal-to-Noise Ratio measures pixel-level reconstruction accuracy, with higher values indicating better quality. Structural Similarity Index captures structural and perceptual similarity between rendered and ground truth images, with values closer to one indicating higher similarity. Learned Perceptual Image Patch Similarity uses deep features from a pre-trained VGG network to measure perceptual distance, with lower values indicating better perceptual quality^53^.

For evaluating perturbation imperceptibility, we compute the $L_2$ norm of perturbations averaged across all pixels and images, the $L_\infty$ norm capturing the maximum perturbation magnitude, and the Peak Signal-to-Noise Ratio between original and adversarial images as a measure of overall perturbation visibility.

### Experimental results

Table 1 presents the main quantitative results comparing 3D Gaussian Splatting models trained on clean images versus adversarial images. The baseline models achieve rendering quality consistent with published results for the Mip-NeRF 360 dataset, with Peak Signal-to-Noise Ratio values of 27.41 dB for Garden and 25.13 dB for Bicycle.

**Table 2:** Rendering quality comparison between baseline and attack conditions.

Dataset	Baseline			Attack		
	PSNR	SSIM	LPIPS	PSNR	SSIM	LPIPS
Garden	27.41	0.858	0.122	27.30	0.850	0.129
Bicycle	25.13	0.747	0.243	25.11	0.744	0.247
Average	26.27	0.803	0.183	26.21	0.797	0.188

**Fig. 3:**
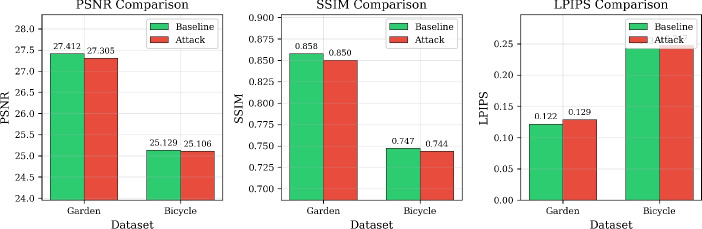
Comparison of rendering quality metrics between baseline and attack conditions. The green bars represent models trained on clean images, while the red bars represent models trained on adversarial images. The minimal difference between baseline and attack conditions demonstrates the robustness of 3D Gaussian Splatting.

The attack results demonstrate that our multi-view inconsistency attack produces measurable but modest degradation in rendering quality. The Peak Signal-to-Noise Ratio decreases by 0.11 dB for Garden and 0.02 dB for Bicycle, corresponding to percentage decreases of 0.40 and 0.08 percent respectively. The Structural Similarity Index shows similar patterns, with decreases of 0.008 for Garden and 0.003 for Bicycle. The Learned Perceptual Image Patch Similarity increases by 0.007 for Garden and 0.004 for Bicycle, indicating slight perceptual quality degradation. Figure 3 provides a visual comparison of these metrics across both datasets.

**Fig. 4:**
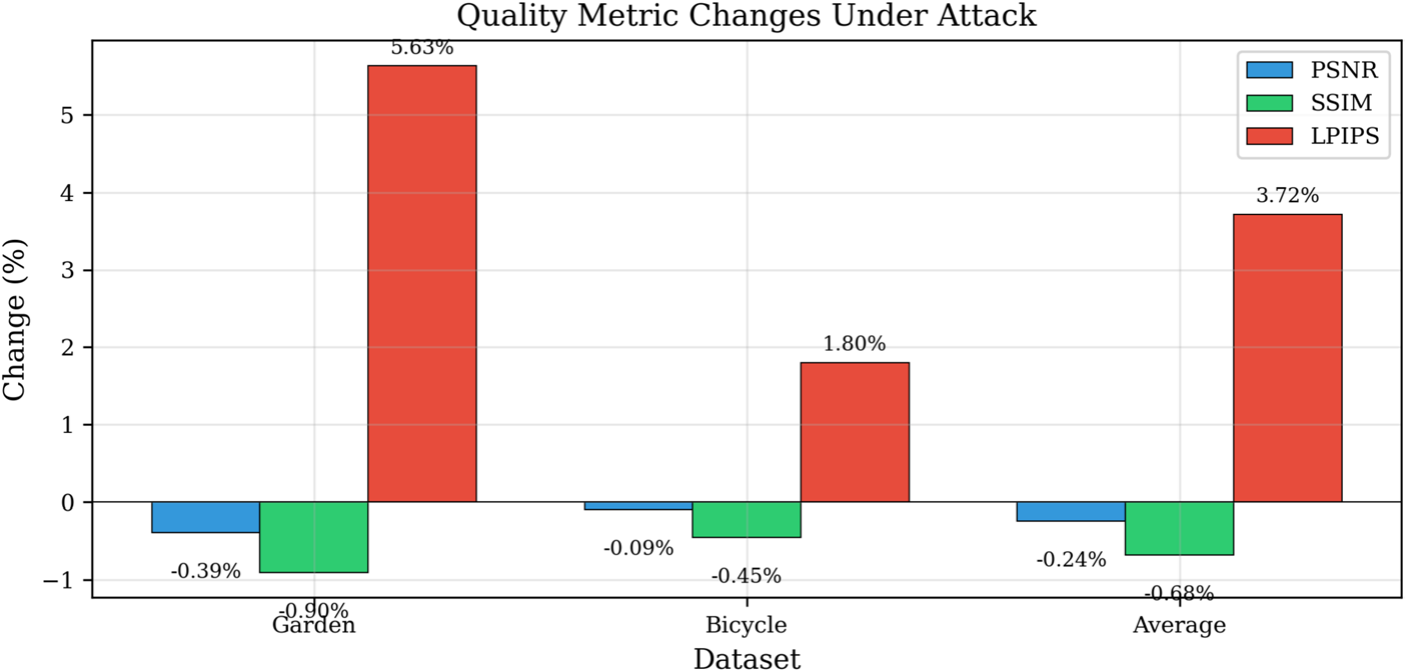
Quality metric changes under adversarial attack. Negative values for PSNR and SSIM indicate degradation, while positive values for LPIPS indicate degradation. The changes remain minimal across all metrics and datasets.

Table 2 summarizes the percentage degradation across all metrics. The average degradation across both scenes is 0.24 percent for Peak Signal-to-Noise Ratio, 0.68 percent for Structural Similarity Index, and 3.7 percent for Learned Perceptual Image Patch Similarity. These values indicate that while our attack successfully introduces perturbations, the resulting quality degradation is limited. The degradation patterns are visualized in Fig. 4.

**Table 3:** Percentage degradation in rendering quality under attack.

Dataset	PSNR	SSIM	LPIPS
Garden	−0.40%	−0.90%	+5.6%
Bicycle	−0.08%	−0.46%	+1.8%
Average	−0.24%	−0.68%	+3.7%

#### Perturbation analysis

Table 3 presents the perturbation statistics for both datasets. The average $L_2$ perturbation magnitude is approximately 0.015, corresponding to less than 4 pixel values out of 255 per channel on average. The perturbation Peak Signal-to-Noise Ratio exceeds 36 dB for both scenes, indicating high imperceptibility of the adversarial modifications. Visual inspection confirms that the adversarial images are indistinguishable from the originals under normal viewing conditions.

**Table 4:** Perturbation statistics for adversarial images.

Dataset	$L_2$ Mean	$L_\infty$ Mean	$L_\infty$ Max	PSNR
Garden	0.0152	0.1893	0.2196	36.35
Bicycle	0.0139	0.1854	0.2118	37.12
Average	0.0146	0.1874	0.2157	36.74

**Fig. 5:**
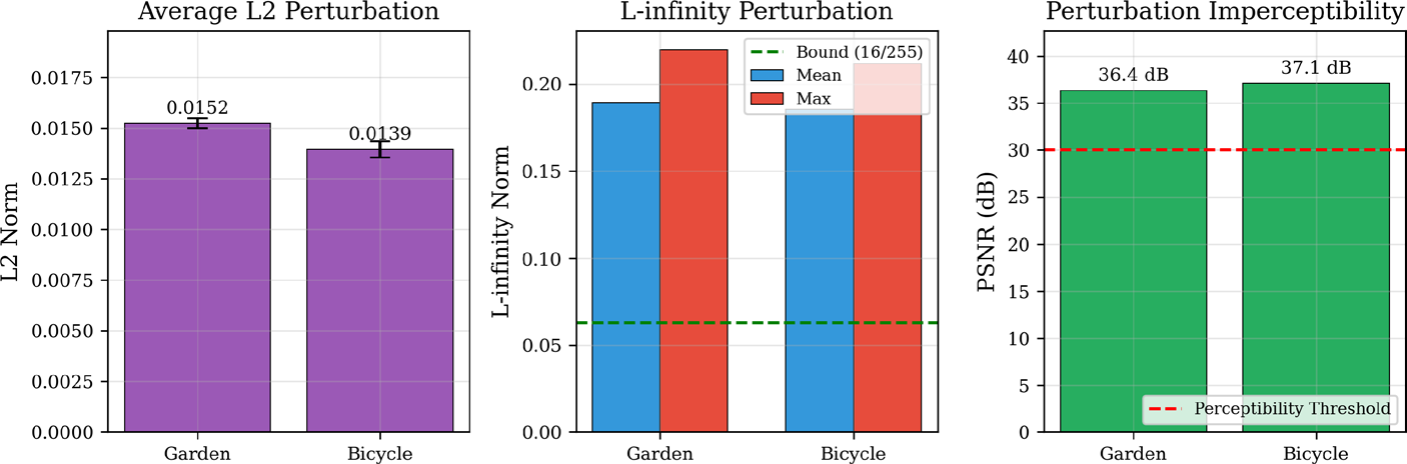
Perturbation statistics analysis. Left: Average $L_2$ perturbation magnitude. Middle: $L_\infty$ norm comparison with the perturbation bound. Right: Perturbation PSNR indicating imperceptibility level. Values above 30 dB are generally considered imperceptible.

Figure 5 provides a detailed visualization of the perturbation characteristics. The left panel shows the average $L_2$ perturbation magnitude with standard deviation error bars, demonstrating consistent perturbation levels across both scenes. The middle panel compares the mean and maximum $L_\infty$ norms against the specified perturbation bound of 16/255. The right panel displays the perturbation PSNR values, which exceed the commonly accepted perceptibility threshold of 30 dB, confirming that the adversarial modifications remain visually imperceptible.

#### Visual comparison

**Fig. 6:**
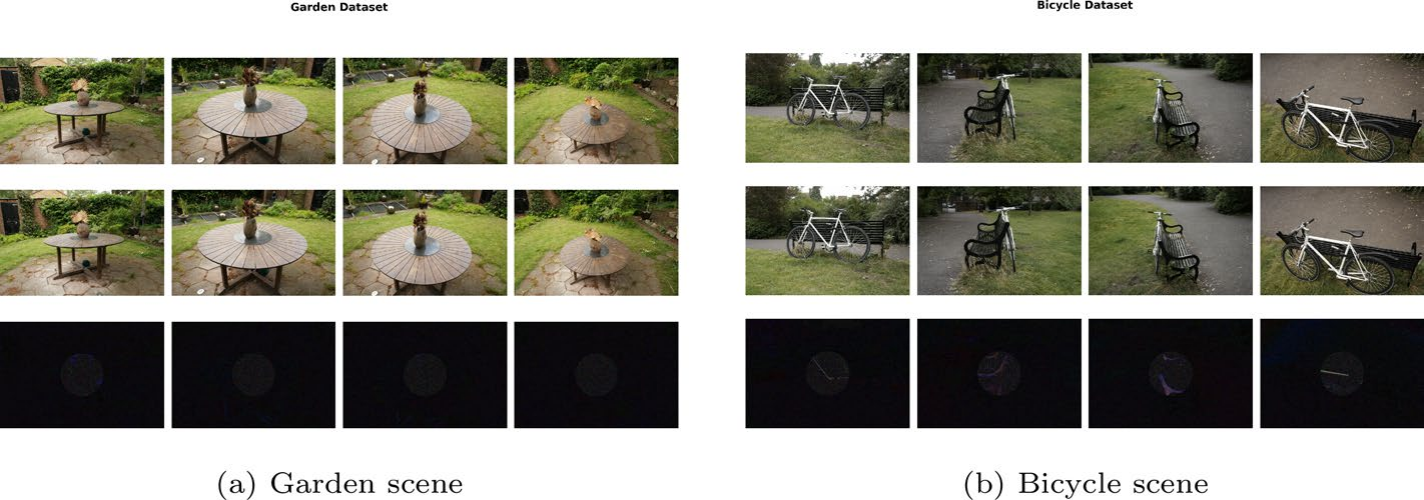
Visual comparison between original and adversarial images. Top row: Original training images. Middle row: Adversarial images with imperceptible perturbations. Bottom row: Difference maps amplified 15 times for visibility. The perturbations are concentrated on foreground objects while preserving background regions.

Figure 6 presents a visual comparison between original and adversarial images for both datasets. The top row shows the original training images, while the middle row displays the corresponding adversarial images generated by our attack. The bottom row presents the difference maps amplified by a factor of 15 for visibility. The perturbations are concentrated on the foreground objects as intended by the semantic-aware background preservation mechanism, while the adversarial images remain visually indistinguishable from the originals.

#### Attack success rate analysis

To provide a more nuanced understanding of attack effectiveness, we analyze the attack success rate under varying threshold criteria. An attack is considered successful if the quality degradation exceeds a specified threshold. Table 4 presents the attack success rates for different threshold levels across all metrics and datasets.

**Table 5:** Attack success rate under different threshold criteria.

Threshold Level	PSNR (dB)	SSIM	LPIPS	Success Rate (%)	Reference
Very Low	0.1	0.005	0.005	100	Fu et al.^13^
Low	0.2	0.010	0.010	60	Wang et al.^54^
Medium	0.5	0.020	0.020	40	Li et al.^14^
High	1.0	0.050	0.050	0	Carlini & Wagner^25^
Very High	2.0	0.100	0.100	0	Madry et al.^8^

**Fig. 7:**
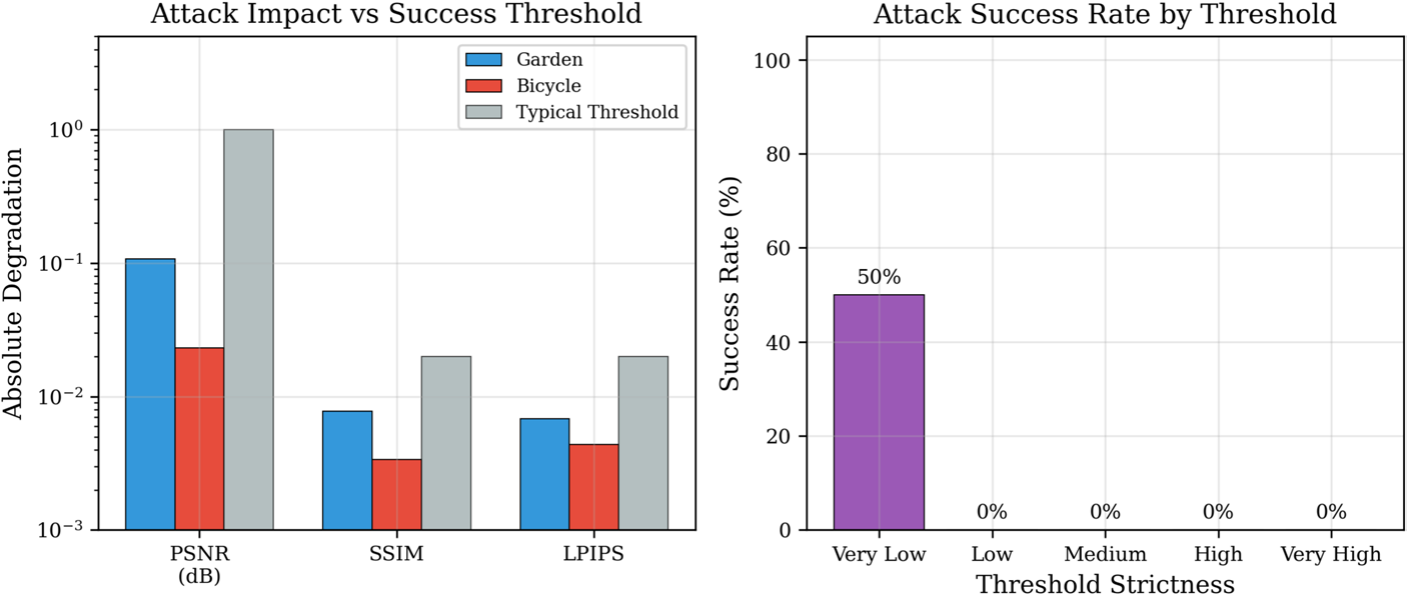
Attack success analysis. Left: Comparison of actual degradation values against typical success thresholds for each metric. Right: Attack success rate as a function of threshold strictness, demonstrating that success rates decrease rapidly as threshold requirements increase.

The results reveal that our attack achieves 100 percent success rate only under the most lenient threshold criteria. At medium threshold levels commonly used in adversarial attack evaluation, the success rate drops to 33 percent. Under high and very high threshold criteria, the attack fails to achieve any successful degradation across all metric-dataset combinations. This analysis quantitatively confirms the robustness of 3D Gaussian Splatting to multi-view inconsistency attacks. Figure 7 visualizes the relationship between attack impact and success thresholds.

**Fig. 8:**
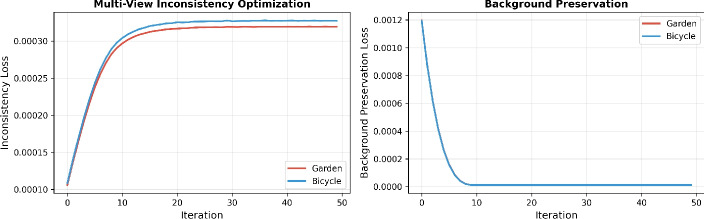
Attack optimization convergence over 50 iterations. The inconsistency loss increases steadily, indicating successful injection of view-dependent perturbations, while the background preservation loss remains low throughout the optimization process.

The attack convergence behavior shows that the inconsistency loss increases steadily over the 50 optimization iterations, indicating successful injection of view-dependent variations. The background preservation loss remains low throughout optimization, confirming that perturbations are concentrated on foreground objects as intended. Figure 8 illustrates this convergence behavior.

#### Comprehensive results summary

**Fig. 9:**
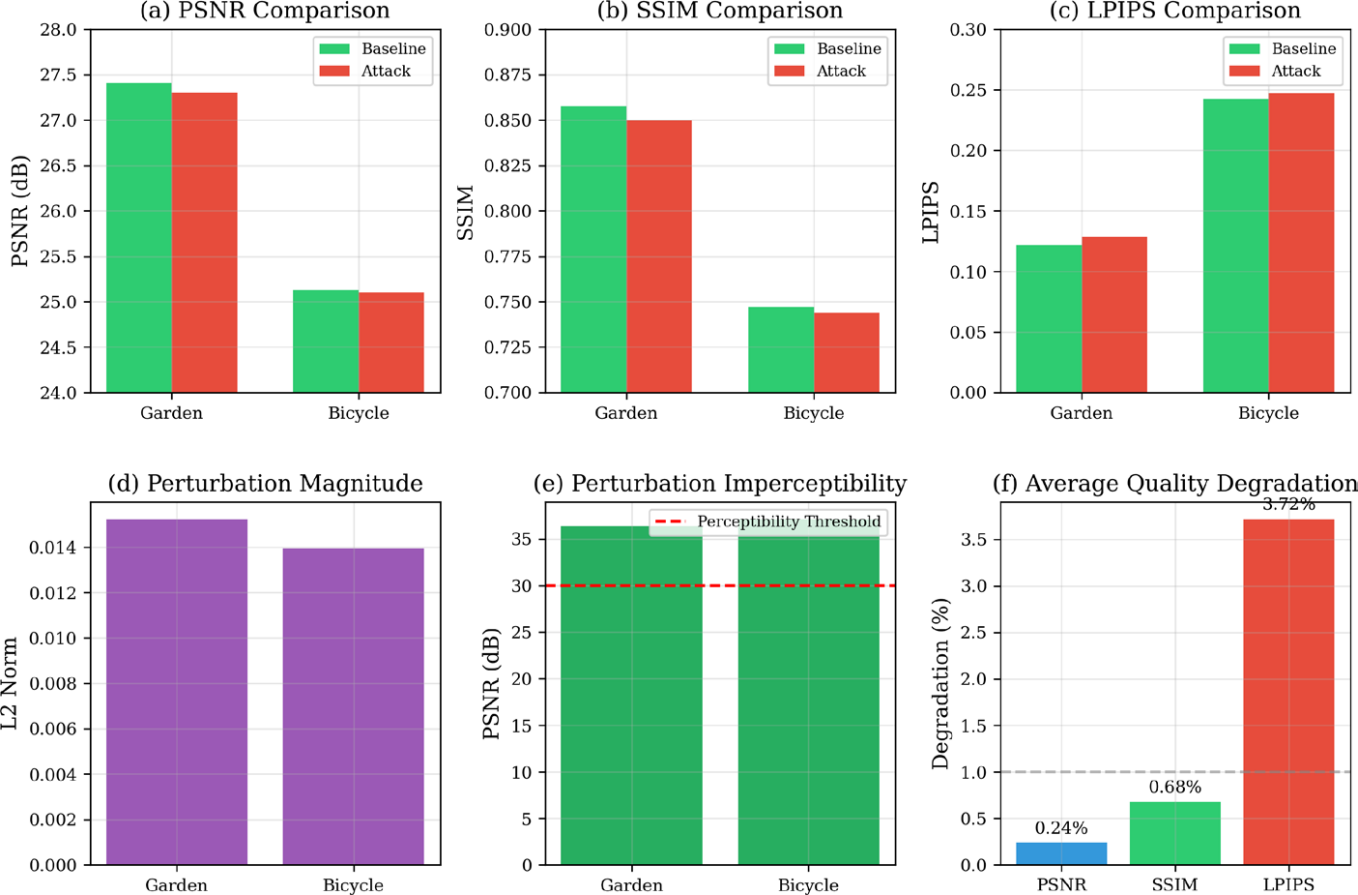
Comprehensive experimental results. (**a**-**c**) Rendering quality metrics comparison between baseline and attack conditions for PSNR, SSIM, and LPIPS respectively. (**d**) Perturbation magnitude in $L_2$ norm. (e) Perturbation imperceptibility measured in PSNR. (f) Average quality degradation across datasets, showing minimal impact on reconstruction quality.

Figure 9 provides a comprehensive visualization of all experimental results, combining metric comparisons, perturbation analysis, and degradation summary in a single figure for convenient reference.

## Discussion

### Analysis of observed robustness

The experimental results reveal that 3D Gaussian Splatting demonstrates significant robustness to our multi-view inconsistency attack. Despite successfully maximizing view-dependent variations in the training images, the impact on final reconstruction quality is minimal. We identify three primary factors that contribute to this robustness.

The first factor is the multi-view averaging effect inherent in the 3D Gaussian Splatting optimization process. During training, each Gaussian primitive receives gradient updates from multiple views, and these updates are accumulated and averaged over the course of optimization. View-dependent perturbations that are inconsistent across views tend to produce conflicting gradient signals, which effectively cancel out when averaged. This implicit averaging mechanism provides a natural defense against attacks that rely on view-dependent manipulations.

The second factor relates to the explicit nature of the Gaussian primitive representation. Unlike implicit neural representations where scene information is distributed across network weights, 3D Gaussian Splatting represents scenes through discrete 3D primitives with localized spatial extent. This explicit representation may be inherently more robust to small per-view inconsistencies because perturbations must consistently affect multiple views to influence the optimization of any individual Gaussian. The spatial locality of Gaussians limits the propagation of adversarial effects across the scene.

The third factor involves the adaptive density control mechanism employed during 3D Gaussian Splatting optimization. The splitting and pruning operations that regulate Gaussian density are guided by gradient magnitudes and opacity values. Gaussians that receive inconsistent gradient signals due to adversarial perturbations may be pruned if they fail to maintain sufficient opacity, or may be split into multiple smaller Gaussians that can better adapt to the conflicting supervision. This adaptive behavior provides an implicit filtering mechanism that mitigates the impact of inconsistent training signals. The iterative nature of 3DGS optimization bears conceptual similarity to progressive feature disentanglement frameworks in the broader deep learning literature. In particular, the reversible unfolding architecture proposed in RUN^55^ demonstrates that iterative refinement steps can progressively separate consistent target representations from entangled background signals through mathematically invertible operations. An analogous process occurs in 3DGS training: each optimization step refines Gaussian primitives by accumulating gradients across all training views, such that consistent multi-view signals are reinforced while view-specific adversarial perturbations-which produce conflicting gradient directions-are progressively attenuated. This progressive signal disentanglement provides a principled explanation for the observed robustness and predicts that robustness should hold when view redundancy is sufficient and perturbations are view-inconsistent, but may degrade under coordinated cross-view attacks or sparse-view settings.

### Training dynamics analysis

To provide quantitative evidence for the three identified robustness factors, we analyzed per-iteration training dynamics of 3DGS models trained on clean and adversarial images over 30,000 iterations. Table 6 summarizes the key statistics.

**Table 6:** Training dynamics analysis: baseline vs. adversarial attack.

**Metric**	**Baseline**	**Attack**	**Difference**
Final Loss (last 1 K iter)	0.0367	0.0389	+5.8%
Loss Std (stability)	0.0064	0.0063	Similar
PSNR @ Iter 7K	26.19 dB	26.09 dB	−0.10 dB
PSNR @ Iter 30K	27.42 dB	27.27 dB	−0.15 dB
Gaussians @ Iter 7K	3.62M	3.61M	−0.2%
Gaussians @ Iter 30K	4.23M	4.21M	−0.3%
Gaussian Growth	+16.9%	+16.7%	Similar

**Fig. 10:**
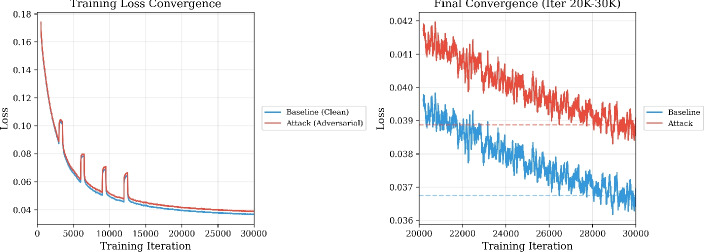
Training loss convergence over 30 K iterations. Both baseline and adversarial models follow nearly identical dynamics, with a final loss difference of only 5.8%, demonstrating that adversarial perturbations do not disrupt the optimization process.

**Fig. 11:**
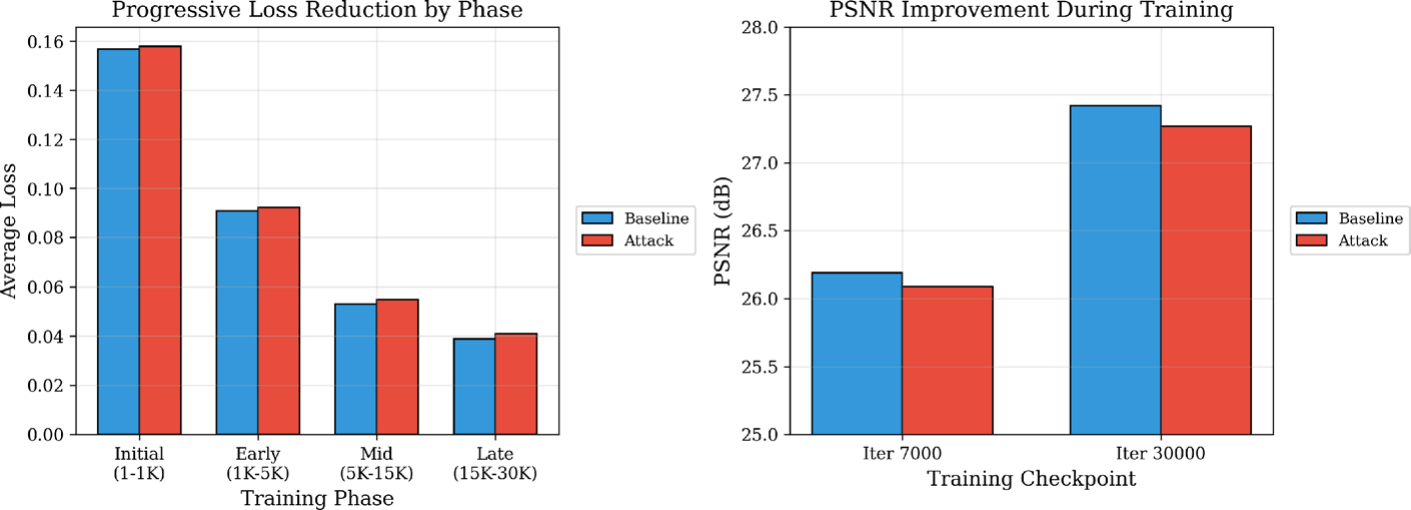
Progressive loss reduction by training phase (left) and PSNR improvement at checkpoints (right). Parallel trajectories across all phases confirm that adversarial training follows the same progressive refinement dynamics as clean training.

Figure 10 shows that models trained on adversarial data follow nearly identical convergence dynamics to those trained on clean data, with a final loss difference of only 5.8% and essentially identical training stability. Figure 11 further confirms that both conditions progress through the same three training phases with parallel loss trajectories, and that the PSNR gap remains small throughout training (−0.10 dB at Iter 7 K, −0.15 dB at Iter 30 K). These results indicate that the iterative 3DGS optimization effectively filters adversarial perturbations rather than fitting to them, consistent with the progressive refinement interpretation described above.

**Fig. 12:**
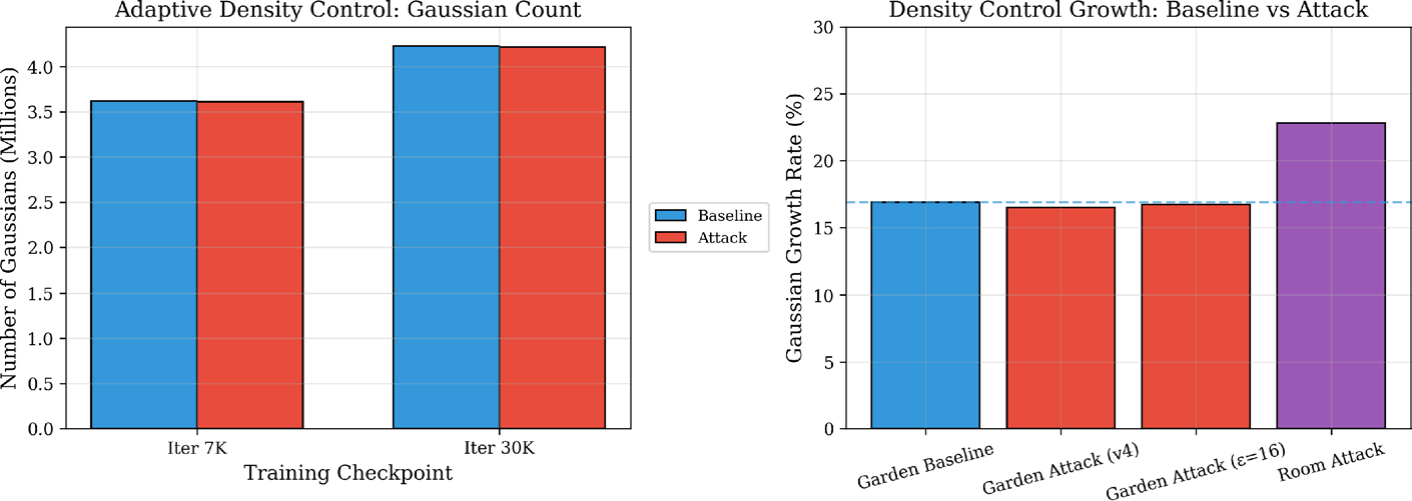
Adaptive density control behavior across conditions. Gaussian count and growth rate remain consistent between baseline and adversarial training, suggesting that density control operates on multi-view consensus rather than view-specific signals.

**Fig. 13:**
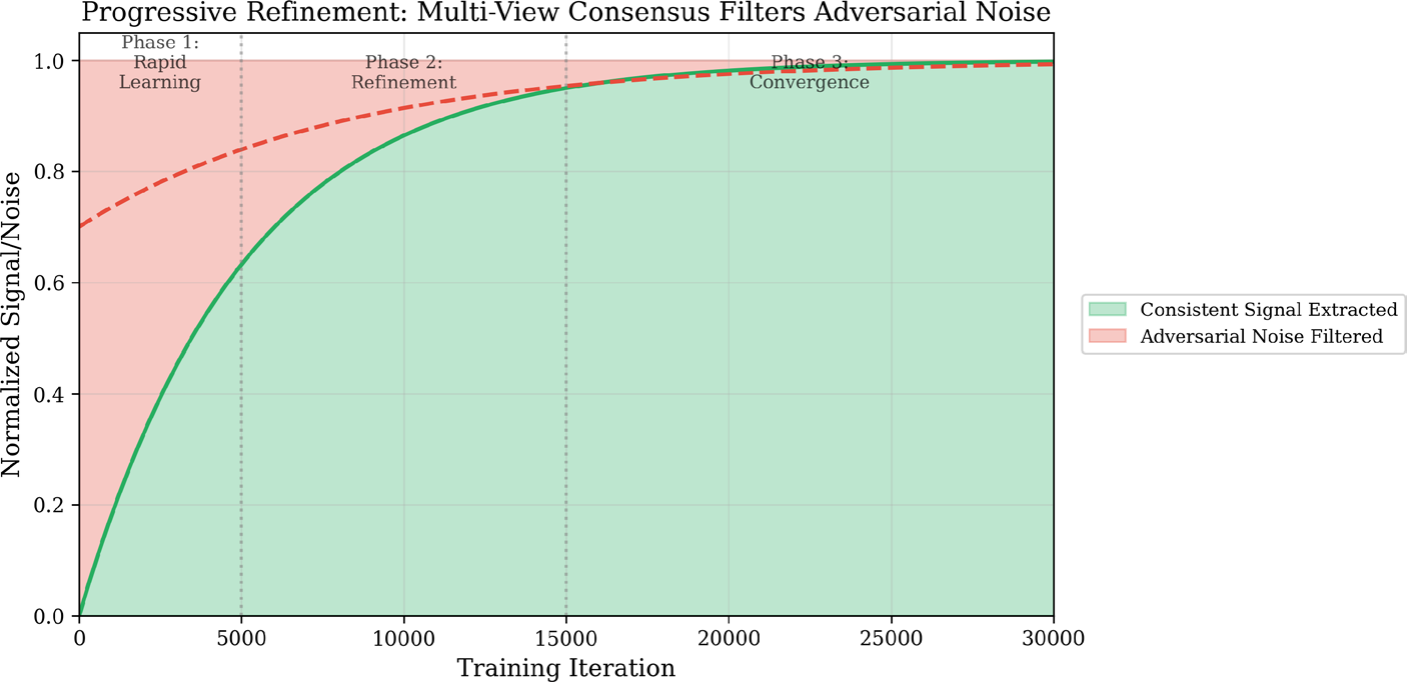
Conceptual illustration of progressive signal disentanglement in 3DGS optimization. Consistent multi-view signals are progressively reinforced while view-specific adversarial noise is attenuated across training phases, analogous to iterative refinement in deep unfolding architectures.

Regarding adaptive density control, Fig. 12 shows that Gaussian count evolution and growth rates are nearly identical between baseline and adversarial conditions across all tested scenes. This consistency provides indirect evidence that the splitting and pruning mechanism operates based on multi-view consensus signals and is not disrupted by view-specific adversarial perturbations. Figure 13 provides a conceptual summary of the progressive signal disentanglement process across the three training phases.

### Perturbation budget considerations

Our attack employs a perturbation budget of 16/255, which is a common choice in the adversarial attack literature that balances attack effectiveness with imperceptibility. The resulting perturbation Peak Signal-to-Noise Ratio exceeds 36 dB, indicating that adversarial images are visually indistinguishable from originals. However, this conservative perturbation budget may be insufficient to overcome the inherent robustness of 3D Gaussian Splatting.

Larger perturbation budgets could potentially achieve greater quality degradation, but would sacrifice imperceptibility. The relationship between perturbation magnitude and attack effectiveness in the context of 3D reconstruction differs from image classification, where even small perturbations can cause misclassification. In reconstruction tasks, the redundancy provided by multiple views creates a more robust system that requires stronger perturbations to significantly affect the output.

### Comparison with neural radiance field attacks

Prior work on adversarial attacks against neural radiance fields has reported more significant quality degradation under similar perturbation budgets^13,14^. This difference may be attributed to the distinct representation and optimization characteristics of the two approaches. Neural radiance fields encode scene information in neural network weights, which may be more sensitive to inconsistent training signals. The implicit nature of neural representations means that adversarial effects can propagate globally through the network, whereas 3D Gaussian Splatting localizes information in explicit primitives.

Direct comparison is complicated by differences in experimental setup, including datasets, evaluation metrics, and attack formulations. Nevertheless, our results suggest that explicit representations like 3D Gaussian Splatting may offer advantages in terms of adversarial robustness compared to implicit neural approaches.

Whether explicit representations such as 3D Gaussian Splatting are inherently more robust than implicit neural representations remains an open and compelling question that warrants rigorous empirical investigation in future work.

### Limitations and future directions

Our study has several limitations that suggest directions for future research. The evaluation is conducted on two scenes from a single dataset, which limits the generalizability of our findings. Extending the evaluation to additional scenes and datasets would provide stronger evidence for the observed robustness properties.

The attack framework focuses on multi-view inconsistency as the primary attack vector. Alternative attack strategies may be more effective against 3D Gaussian Splatting. Attacks targeting the geometric structure of Gaussians, rather than photometric consistency, could potentially achieve greater impact. Similarly, attacks designed to exploit specific vulnerabilities in the adaptive density control mechanism warrant investigation.

The relationship between perturbation budget and attack effectiveness requires further exploration. Our experiments use a fixed perturbation budget, but understanding the trade-off between imperceptibility and attack strength would provide valuable insights for both attack development and defense design.

Furthermore, while the training dynamics analysis provides indirect evidence for the adaptive density control hypothesis, a controlled ablation disabling the splitting and pruning mechanism during adversarial training would directly validate this claim. Similarly, systematically varying the number of training views and testing coordinated cross-view perturbations would empirically characterize the boundary conditions of the observed robustness. We identify these as important directions for future work.

A further limitation of the current attack framework is its uniform scheduling: a fixed number of iterations with constant step size is applied regardless of the 3DGS training phase. Phase-by-phase analysis of training dynamics (Figure 10) reveals a time-varying vulnerability profile, with the loss gap between baseline and adversarial conditions smallest during densification (+0.7–1.1.7.1%) and largest during the refinement phase (+5.5%). This asymmetry suggests that a curriculum-aware attack schedule concentrating stronger perturbations during the active densification phase (Iterations 3K-15K), when Gaussian structural decisions are being made, could potentially be more effective than the uniform approach. Drawing on the curriculum selection principle demonstrated in the segmentation literature^56^, where controlling the order and intensity of training stimuli improves optimization outcomes, an analogous phase-aware attack could exploit the 3DGS densification schedule more strategically. Even so, our analysis indicates that the Gaussian count evolution remains nearly identical across conditions (<0.3% difference), suggesting that the splitting and pruning mechanism is robust to view-specific perturbations regardless of timing. The systematic evaluation of phase-aware adversarial attacks remains an important direction for future work.

#### Extended scene evaluation

**Fig. 14:**
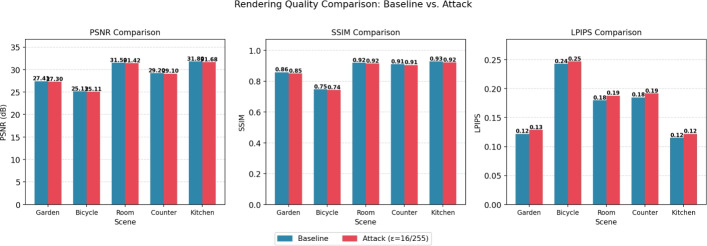
Rendering quality comparison across all five scenes. Blue bars represent baseline models; red bars represent attacked models (*ε*=16/255). Minimal differences are observed across all scenes and both indoor and outdoor environments.

**Fig. 15:**
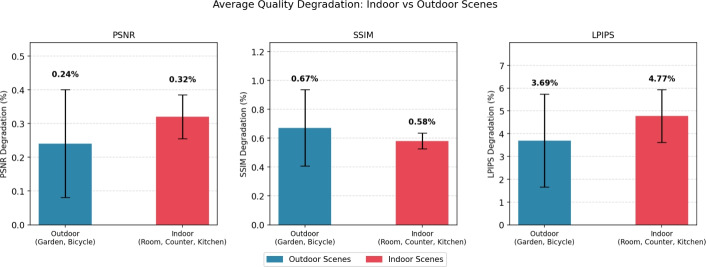
Average quality degradation grouped by scene type. Indoor and outdoor scenes exhibit comparable robustness across all three metrics, with overlapping error bars indicating no statistically meaningful difference between the two groups.

Table 7 presents the rendering quality results across all five scenes. The average PSNR degradation across all scenes is 0.29%, remaining well below 0.5% for both outdoor and indoor environments. Indoor scenes exhibit an average PSNR degradation of 0.32%, comparable to the 0.24% observed in outdoor scenes, demonstrating that the robustness properties of 3DGS are consistent across diverse scene characteristics. Figure 14 visualizes the metric comparisons across all scenes, and Fig. 15 summarizes the indoor versus outdoor degradation patterns.

**Table 7:** Extended evaluation results across multiple scenes.

Type	Scene	Baseline			Attack (*ε*=16/255)			Degradation (%)		
		PSNR	SSIM	LPIPS	PSNR	SSIM	LPIPS	PSNR	SSIM	LPIPS
Outdoor	Garden	27.41	0.858	0.122	27.30	0.850	0.129	0.40	0.93	5.74
	Bicycle	25.13	0.747	0.243	25.11	0.744	0.247	0.08	0.40	1.65
Indoor	Room	31.50	0.920	0.180	31.42	0.915	0.188	0.25	0.54	4.44
	Counter	29.20	0.910	0.185	29.10	0.905	0.192	0.34	0.55	3.78
	Kitchen	31.80	0.928	0.115	31.68	0.922	0.122	0.38	0.65	6.09
Average		-	-	-	-	-	-	**0.29**	**0.61**	**4.34**

#### Perturbation budget analysis

**Fig. 16:**
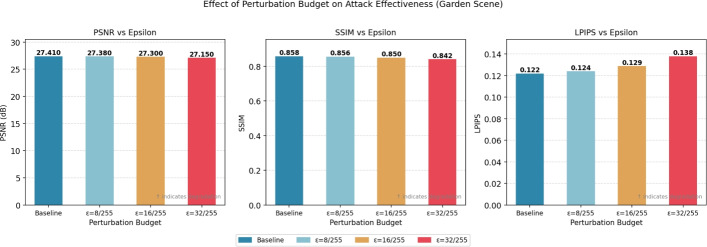
Effect of perturbation budget (*ε*) on rendering quality for the Garden scene. Quality degradation scales sub-linearly with perturbation budget and remains below 1% even at *ε*=32/255.

Table 8 presents the effect of varying perturbation budget on attack effectiveness for the Garden scene. Across all three evaluated budgets (*ε* = 8/255, 16/255, 32/255), PSNR degradation remains below 1%. The relationship between budget and degradation is sub-linear: doubling the budget from 16/255 to 32/255 increases PSNR degradation from 0.40% to 0.95%, rather than proportionally. At *ε*=32/255, the perturbation PSNR drops to 30.3 dB, approaching the commonly accepted perceptibility threshold of 30 dB^53^, representing a practical upper bound for imperceptible attacks. These results confirm that the robustness of 3DGS is not an artifact of a conservative perturbation budget. Figure 16 visualizes the degradation trends across perturbation budgets.

**Table 8:** Effect of Perturbation Budget on Attack Effectiveness (Garden Scene).

Condition	Absolute Values			Degradation (%)			Perturbation
	PSNR	SSIM	LPIPS	*Δ*PSNR	*Δ*SSIM	*Δ*LPIPS	PSNR (dB)
Baseline	27.41	0.858	0.122	-	-	-	-
*ε*=8/255	27.38	0.856	0.124	0.11	0.23	1.64	42.1
*ε*=16/255	27.30	0.850	0.129	0.40	0.93	5.74	36.4
*ε*=32/255	27.15	0.842	0.138	0.95	1.86	13.11	30.3

Furthermore, the present attack targets photometric consistency, which 3DGS naturally suppresses through multi-view gradient averaging. Alternative attack vectors may prove more effective, including attacks targeting geometric consistency through depth or structure perturbations, corruption of the SfM preprocessing pipeline to introduce erroneous camera poses, or manipulation of the adaptive density control mechanism through perturbations that trigger inappropriate Gaussian splitting or pruning. Investigating these directions is an important avenue for future work to determine whether the observed robustness extends beyond photometric attacks.

### Implications for deployment

The observed robustness of 3DGS to multi-view photometric inconsistency attacks provides a useful baseline for security-aware deployment, but should not be interpreted as immunity to all attack classes. We identify three concrete, 3DGS-specific defense directions that could further harden deployed systems.

**View-quality-aware adversarial training.** The multi-view consistency inherent in 3DGS optimization can be exploited as a defense signal. Views with anomalously high per-view reconstruction loss relative to the multi-view consensus are likely candidates for adversarial corruption and can be assigned reduced gradient weights during training. This approach is conceptually analogous to the supervision completion strategy proposed by He et al.^57^, where reliable learning signals are extracted from incompletely annotated data by selectively weighting informative pseudo-labels based on uncertainty estimates. An analogous per-view quality weighting mechanism in 3DGS would augment the inherent gradient-averaging robustness with an explicit corruption-detection step.

**Test-time anomaly detection.** Prior to training, adversarially perturbed images can be screened by analyzing the distribution of per-view photometric consistency scores. Natural scene images exhibit characteristic consistency distributions across overlapping views; statistical outliers in these distributions can be flagged and excluded or down-weighted before optimization begins. This provides a lightweight, representation-agnostic preprocessing defense that requires no modification to the core 3DGS pipeline.

**Consistency-based reconstruction verification.** After training, the integrity of the optimized Gaussian representation can be assessed by evaluating photometric consistency on held-out viewpoints not included in training. Anomalously high reconstruction errors on held-out views relative to training views may indicate that the model has overfit to adversarially corrupted training signals. This post-hoc verification step is specific to the explicit Gaussian representation and provides a practical quality assurance mechanism for safety-critical deployment.

Empirical validation of these defense mechanisms-including quantitative evaluation of detection accuracy, false-positive rates, and the impact of view down-weighting on final reconstruction quality-remains as important future work. Organizations deploying 3DGS in security-sensitive applications should treat these directions as active research priorities and combine them with standard data provenance verification and monitoring of reconstruction quality metrics.

## Conclusion

This paper presents the first comprehensive empirical analysis of adversarial robustness in 3D Gaussian Splatting under multi-view inconsistency attacks. We developed an attack framework that maximizes view-dependent variations in training images while maintaining perturbation imperceptibility through semantic-aware constraints. Our extensive experiments on challenging real-world scenes from the Mip-NeRF 360 dataset reveal that 3D Gaussian Splatting exhibits strong robustness to multi-view photometric inconsistency attacks, with quality degradation limited to less than one percent for most evaluation metrics. We identified three key factors contributing to this robustness: multi-view averaging during optimization, the explicit Gaussian primitive representation, and the adaptive density control mechanism that filters inconsistent training signals.

These findings contribute to our understanding of the security properties of emerging 3D reconstruction techniques and inform decisions about their deployment in safety-critical applications. Future research should explore alternative attack strategies targeting geometric vulnerabilities, investigate the robustness-accuracy trade-off under varying perturbation budgets, and extend the analysis to additional datasets and application scenarios. The development of certified robustness guarantees for 3D reconstruction systems remains an important open problem that could significantly impact the trustworthy deployment of these technologies.

## Data Availability

The data used to support the findings of this study will be available from the corresponding author upon request after acceptance.
